# Guided growth for valgus deformity correction of knees in a girl with osteopetrosis: a case report

**DOI:** 10.1007/s11751-017-0290-x

**Published:** 2017-06-07

**Authors:** Dmitry Popkov

**Affiliations:** Russian Ilizarov Scientific Center for Restorative Traumatology and Orthopaedics, 6, M.Ulyanova Street, Kurgan, Russian Federation 640014

**Keywords:** Osteopetrosis, Genu valgum, Tension band technique hemiepiphysiodesis, Guided growth

## Abstract

Autosomal dominant osteopetrosis (Albers-Schönberg disease) classically displays the radiographic signs of osteosclerosis. The main ADO complications involve the skeleton: low-impact bone fractures, scoliosis and hip osteoarthritis. Management of osteopetrosis-related orthopedic problems is a surgical challenge due to increased bone density. The healing process is very slow in these patients because of bone remodeling defects related to osteoblast function failure. In case of bone deformities, a realignment method should be appropriated to osteopetrosis conditions. This article presents a case report of operative treatment of an 11-year-old girl affected with ADO, who underwent a simultaneous valgus knee deformity correction of both limbs with medial eight-plate epiphysiodesis. Simultaneous correction of valgus deformity on both limbs using an extraperiosteal tension plate technique for medial tibial hemiepiphysiodesis was performed in a girl of 11.5 years old with autosomal dominant osteopetrosis. The treatment duration from surgery to complete deformity correction and removal of plates was 18 months. The final aMPTA was 86° on the right side and 85° on the left side. The correction rate was 0.61°/month (right tibia) and 0.67°/month (left tibia). The MAD correction rate was evaluated as 1.5 mm/month for the right limb and 1.6 mm/month for the left limb. At the moment of plate removal, one screw was broken because of tight fixation in osteopetrotic bone. But it did not compromise the final result. The latest follow-up visit at the age of 14 years 6 months revealed excellent realignment without any deformity relapse. There was no any functional impairment. We consider the guided growth by tension band technique as very interesting and promising solution for treatment of pediatric angular deformity in patients with OP. This method allows to avoid osteotomy and related important risk of delayed union or nonunion frequently observed in osteopetrosis.

Level of evidence: Level IV.

## Introduction

Osteopetrosis (OP) comprises a set of rare clinically heterogeneous diseases characterized by increased bone mass [1–3]. The increase in bone density results from defective osteoclast differentiation or function [1, 3]. The incidence of OP is difficult to estimate. Autosomal recessive osteopetrosis (ARO) has an incidence of 1–3.4 in 250,000 live births [4–6]. Overall incidence is 1 in 20,000 births for autosomal dominant osteopetrosis (ADO) [5, 6].

ARO presents the most severe forms diagnosed in the first months of life [1, 7, 8]. The increase in bone density weakens the bone, resulting in predisposition to fractures and osteomyelitis. The impaired longitudinal growth and short stature are classical for the ARO. Children have typical facial appearance (macrocephaly and frontal bossing). The skull changes can lead to hydrocephalus and hindbrain posterior fossa crowding [7]. Pancytopenia because of bone marrow suppression is the most severe complication of ARO [1]. Cranial nerve compressions can result in blindness, deafness and palsy. In severe forms, the life expectancy without hematopoietic stem cell transplantation is reduced: Untreated children die in the first decade as a complication of bone marrow suppression [9–11].

ADO has late onset in childhood or adolescence. In contrast to ARO, the life expectancy in these forms is regular [1, 12–14]. In ADO, the genetic mutation in the chloride channel 7 gene (CLCN7) is detected in about 70% of patients [15]. Albers-Schönberg disease classically displays the radiographic signs of osteosclerosis at the vertebral level, a bone in bone aspect, alternating dense and light bands [1, 2, 12]. The main ADO complications involve the skeleton: bone fractures (with mean of 3 fractures per patient), scoliosis, hip arthritis, mandibular osteomyelitis and dental abscess [1, 13, 14, 16]. On the other hand, cranial nerve compressions and short stature are rare [1, 14]. Management of osteopetrosis-related fractures presents a surgical challenge due to increased bone density [14, 17]. Furthermore, the healing process is very slow in these patients because of bone remodeling defects related to osteoblast function failure (low rate of bone turnover) [2, 18, 19]. Nonunion or delayed bone union might occur [14, 17, 19, 20]. There are a lot of case reports regarding the treatment of osteopetrosis-related fractures in references [13, 14, 16, 20–23]. But in bone deformities due to impaired growth or malunion of a fracture, realignment methods should be appropriated to osteopetrosis conditions as well. We did not find any study or case report published about deformity correction of lower limbs in children with osteopetrosis. Only one paper concerning the result of a case of lower limb length discrepancy correction was found [24].

This article presents a case report of operative treatment of an 11-year-old girl affected with ADO, who underwent a simultaneous valgus knee deformity correction of both limbs with medial eight-plate epiphysiodesis.

## Materials and methods

In our institution, a girl of 11 years 1 month old (height 144 cm, weight 37 kg) was examined because of valgus deformity of the knee and a year history of moderate anterior knee pain related to physical activity. Symmetric valgus knee deformity had gradually been progressing for recent 18 months according to the parent’s opinion. Bilateral genu valgum caused circumduction gait.

**Fig. 1:**
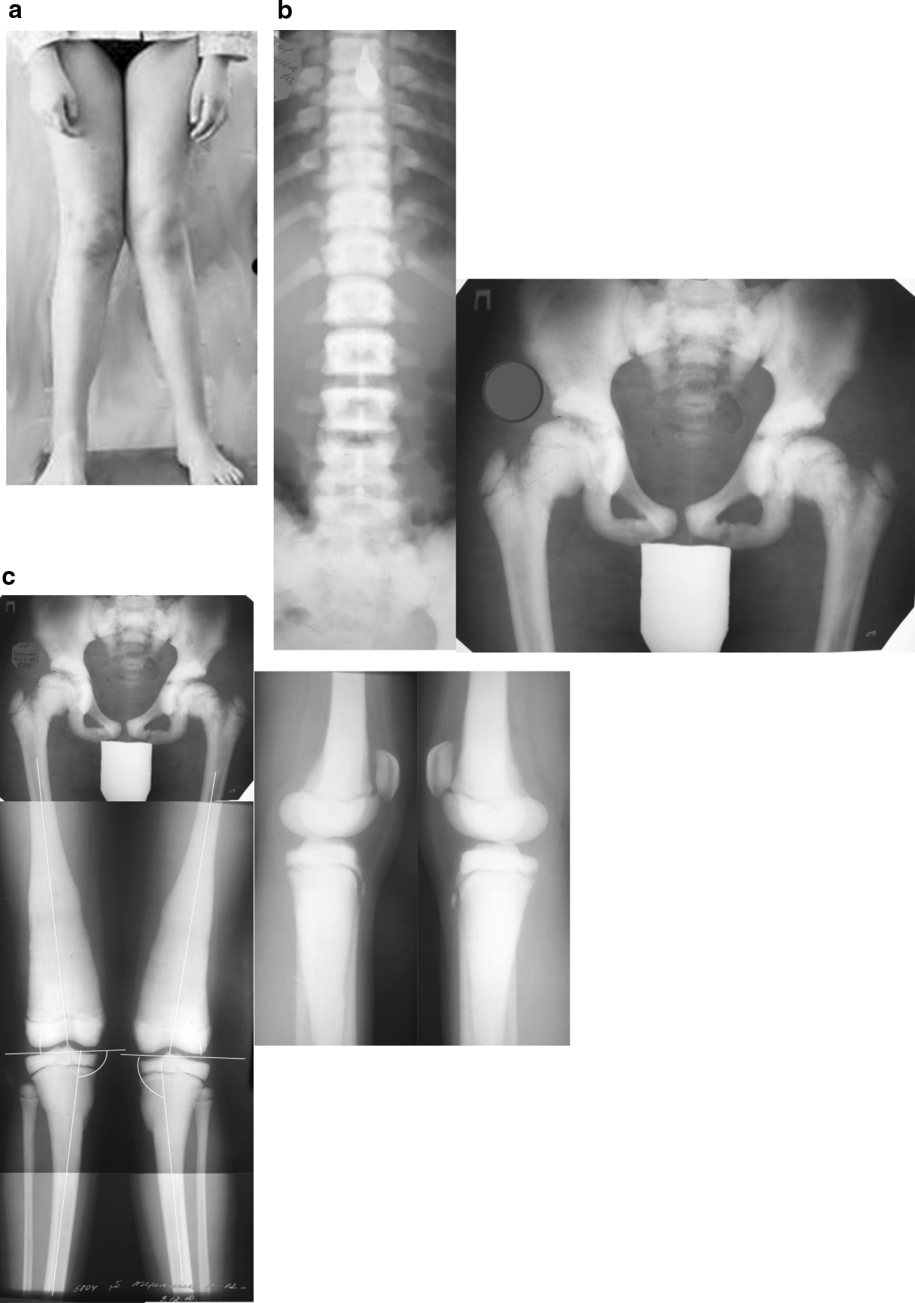
Before surgery: **a** aspect of lower limbs; **b** AP radiographs of the spine and pelvis, note increased bone density; **c** radiographs of lower limbs: aLDFA 84° (*right side*) and 82° (*left side*); aMPTA 97° for both tibias

During a physical examination, the patient had evident valgus deformity of both knees (Fig. 1). Range of motion in knees, hips and ankles was not limited. But within the latest year, a walking distance did not exceed 2 km. Ocular examination revealed slight bilateral vision loss. Other systemic examinations showed normal vital findings. Laboratory tests were within normal limits, except mild anemia (erythrocytes 3.9 × 10^6^ ml; hemoglobin 108 g/l). Patient’s history revealed two asynchronous femoral neck fractures at the age of 7 years. Conservative management was satisfactory without development of coxa vara (Fig. 1). The family history of the patient did not indicate osteopetrosis. The parents available for examination revealed no manifestation of osteopetrosis.

X-ray examinations revealed diffuse osteosclerosis, affecting spine, pelvis and long bones with bone modeling defects at the metaphyses (Fig. 1). We used several standard criteria to evaluate radiological parameters [25]. The anatomical lateral distal femoral angle (aLDFA) was 84° (right side) and 82° (left side). Values of anatomical medial proximal tibial angle (aMPTA) were pathological and corresponded to valgus deformity: 97° for both tibias. That deformity determined lateral mechanical axis deviation (MAD) of 24 mm on the right limb and 25 mm on the left limb. No deformities were detected in the lateral plane.

**Fig. 2:**
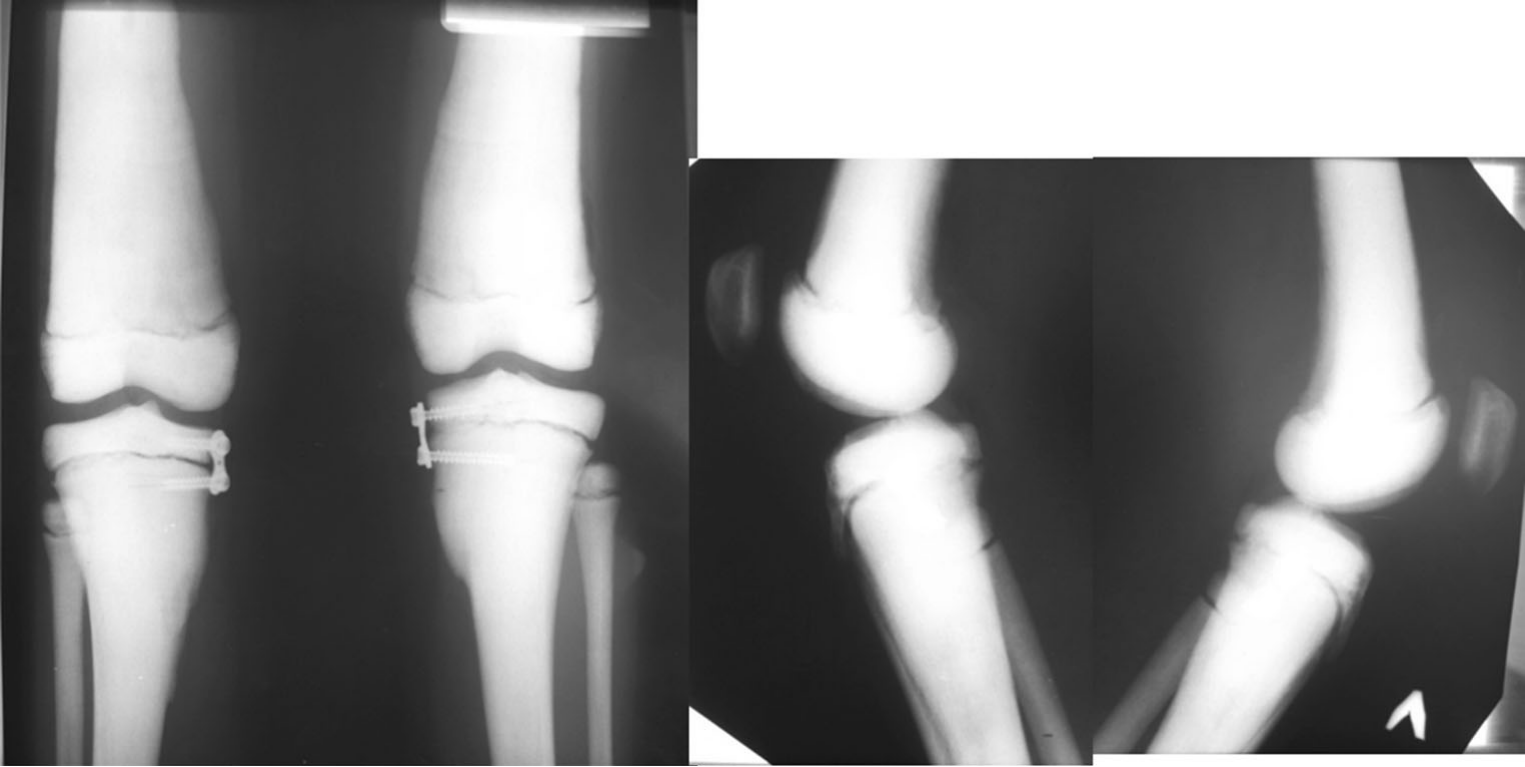
AP and lateral knee radiographs of lower limbs after surgery

For simultaneous correction of valgus deformity on the both limbs, we performed extraperiosteal tension band technique (eight-plate) medial tibial hemiepiphysiodesis in 6 months after the first visit. The guided growth is the most low-invasive method in pediatric deformity correction. It avoids any kind of complications caused by osteotomy and osteosynthesis (Fig. 2).

**Fig. 3:**
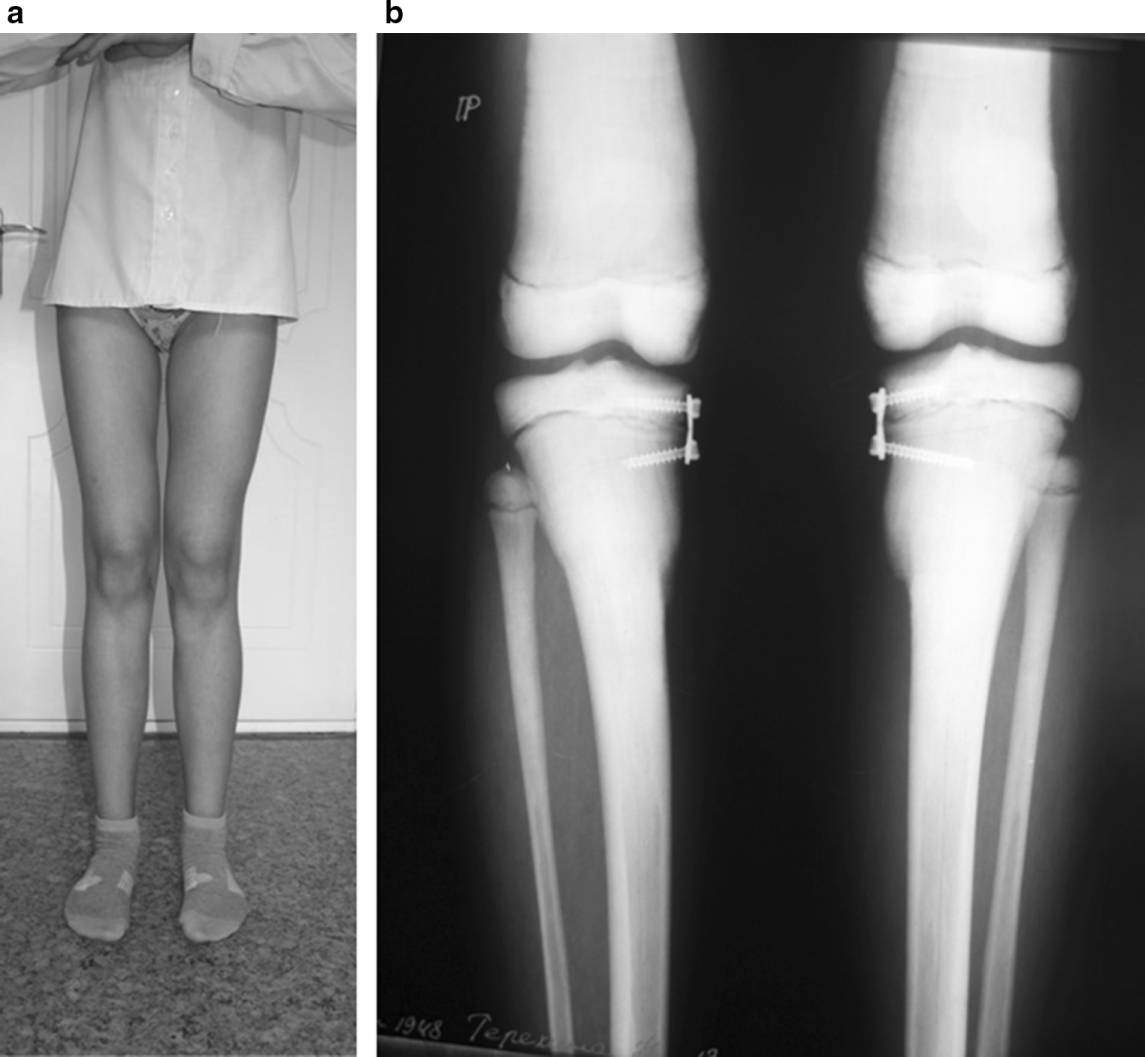
During treatment: **a** lower limb aspect in 9 months after surgery; **b** AP radiographs of lower limbs in 12 months after eight-plate implant

Application of eight plates was performed under general anesthesia via standard approaches. We used slow-speed high-torque electric drills and frequent cooling with physiological saline while preparing canals for screws. No immobilization was required after surgery, and early weight bearing was encouraged with a rapid return to normal activities. Follow-up evaluation (AP weight-bearing X-rays and clinical examination) was performed every 3 months approximately because the rate of angular deformity correction in children with OP is unclear (Fig. 3).

## Results

**Fig. 4:**
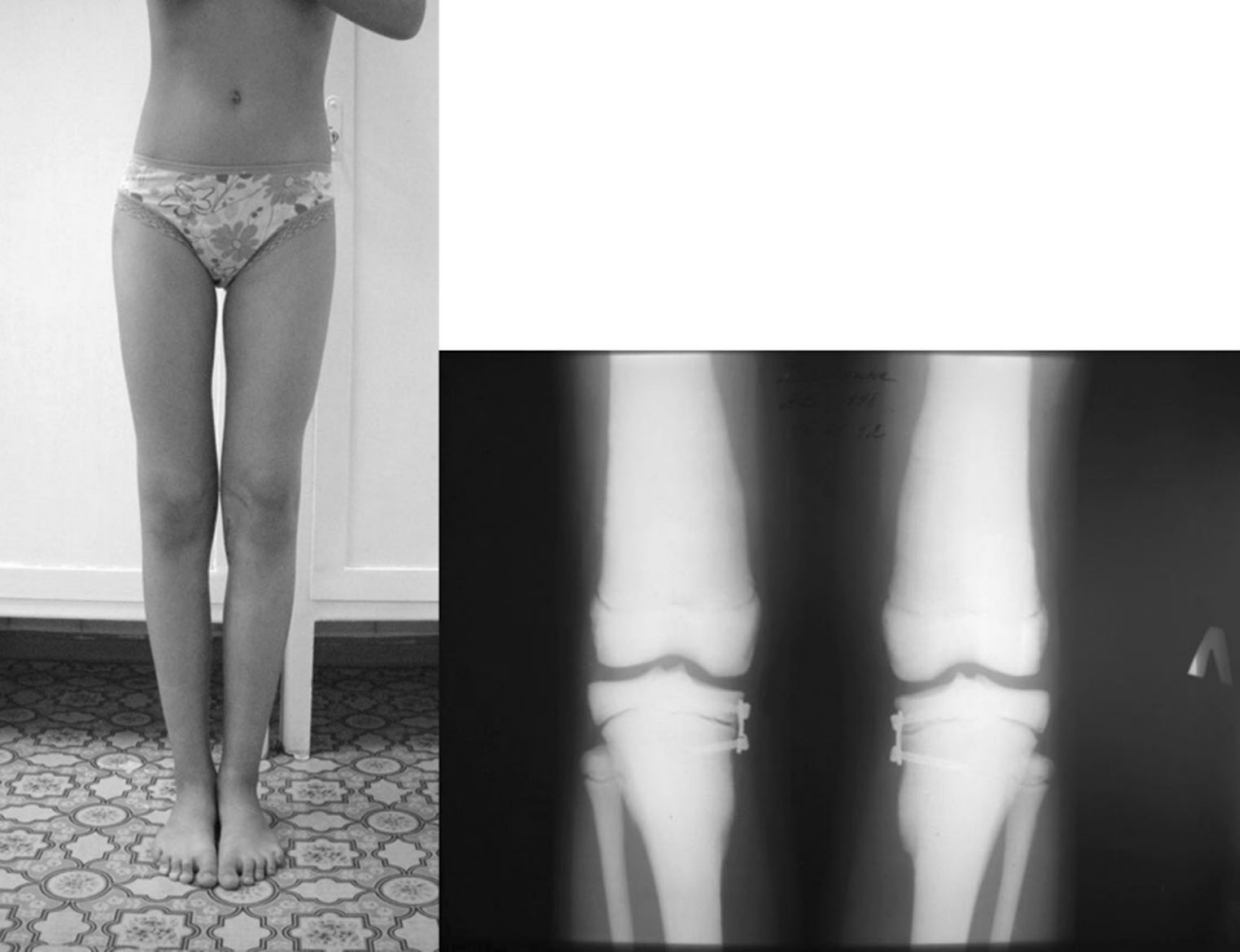
In 18 months after surgery, surgery plates removal is planned

The treatment duration from surgery to removal of plates (realignment achieved) was 18 months. During treatment, we observed progressive clinical and radiological correction of valgus knee deformity. The final aMPTA was 86° on the right side and 85° on the left side so that the degree corrected was 11° for the right tibia and 12° for the left tibia. The correction rate was 0.61°/month (right tibia) and 0.67°/month (left tibia). At the moment of plate removal, the medial MAD corresponded to normal realignment in children of that age [26]: medial position of 3 mm to center of right knee joint and 4 mm medial to center of left knee joint. The MAD correction rate was evaluated as 1.5 mm/month on the right and 1.6 mm/month on the left (Fig. 4).

**Fig. 5:**
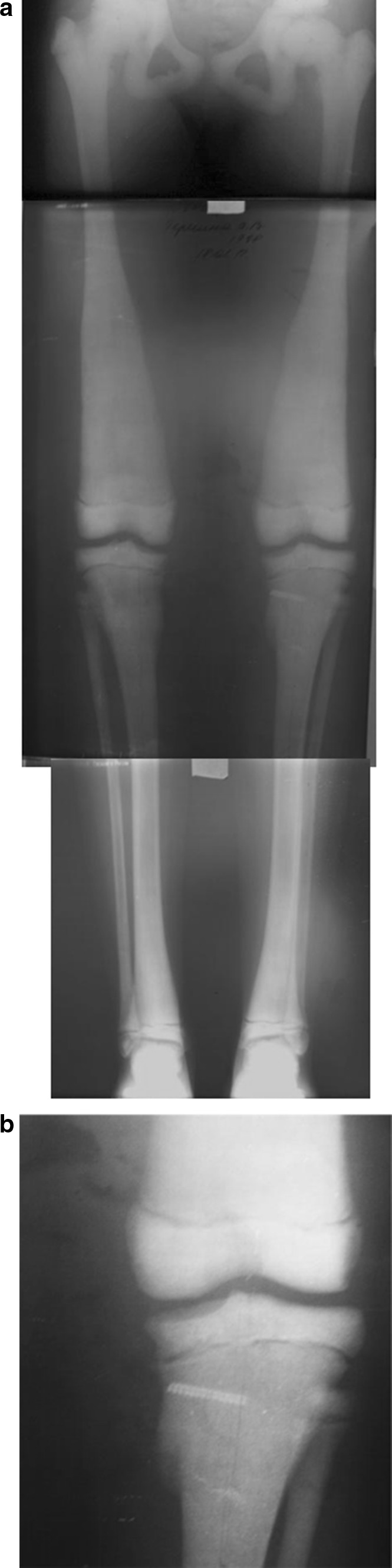
Radiographs after frames removal: **a** aMPTA 86° (*right side*) and 85° (*left side*); *MAD* medial position of 3 mm to center of right knee joint and 4 mm medial to center of left knee joint; **b** broken screw in tibial metaphysis

At the moment of plate removal, one screw was broken because of tight fixation in osteopetrotic bone. But it did not compromise the final result (Fig. 5).

**Fig. 6:**
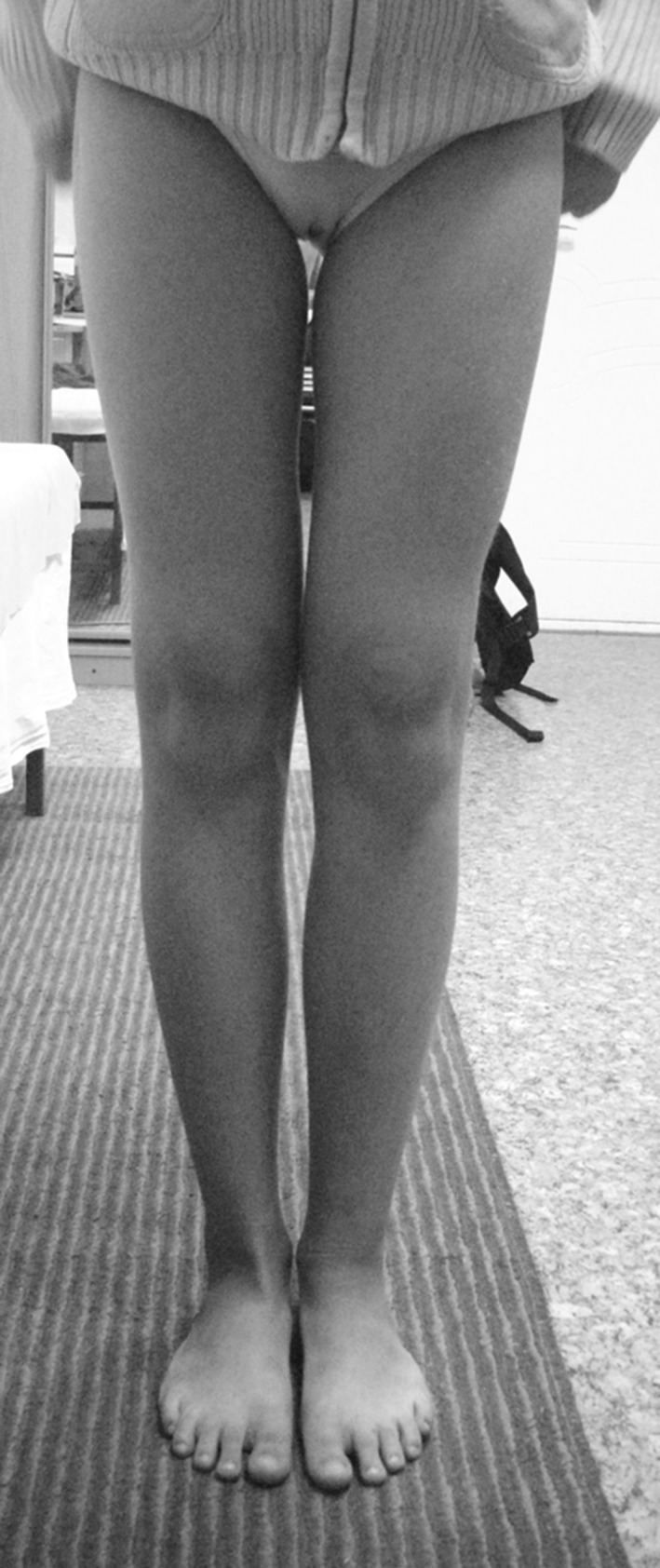
Patient at the age of 14 years 6 months old

At the latest follow-up visit at the age of 14 years 6 months old (menstruation occurred), clinical realignment was excellent without any deformity rebound. There was no any functional impairment. We did not observe the decrease in range of motion in the knee. The patient refused an X-ray control in order to reduce irradiation, and we did not insist because the clinical result was absolutely satisfactory (Fig. 6).

## Discussion

Angular knee deformities alter biomechanics of the limb by causing a distorted stress distribution on the weight-bearing surface of the knee joint [27]. Surgical intervention should be considered for a deformity exceeding 10° with a predicted remaining time for growth of at least 12 months [28]. The eight-plate hemiepiphysiodesis method is often preferred for pediatric angular deformity correction as a low-invasive method to restore lower limb realignment [28–31].

In patients with OP, the increased bone mass is observed due to osteoblast dysfunction [1, 3, 6, 15] and bone fragility—due to abnormal remodeling rate and balance [18, 32]. Arruda et al. [33] found a great heterogeneity in bone microstructure in adult patients with ADO, using X-ray absorptiometry and high-resolution peripheral quantitative computed tomography. They showed that the accumulation of old and fragile bone randomly distributed along the skeleton. These alterations in bone microstructure may contribute to high prevalence of low-impact fractures in patients with ADO [12, 13, 16, 33]. On the other hand, the osteopetrotic bone consolidates with pathological callus without Haversian organization due to osteoclast function failure [34].

The dominant types of OP are more clinically challenging because of normal life expectancy in comparison with recessive (malignant) forms [1, 3, 17, 35, 36]. The main orthopedic problems in children and adults with ADO are low-impact fractures, coxa vara, osteoarthritis and osteomyelitis [12, 14, 18, 19].

The publications about osteopetrotic fractures, especially femoral fractures, and their treatment are common. The majority of colleagues suggest that the osteosynthesis is the primary treatment of choice in management of osteopetrotic femoral fractures [13, 16, 17, 21–23]. But they report several types of complication related to the surgery due to hard-fragile sclerotic bones: bending of drill bits or screws, and osteomyelitis [12, 14]. Delayed union or nonunion of fracture and related implant failure are also reported in the literature [14, 20, 23]. Troubles of bone consolidation in patients with OP are due to remodeling failure of callus [18, 19, 37]. Malunion of femoral fractures can result in coxa vara and develop osteoarthritis of the hip requiring arthroplasty that provides good outcomes [36]. In case of knee osteoarthritis, the results of total knee arthroplasty are also excellent [19].

Very slow bone consolidation and frequently observed implant failures justify the use of the orthopedic methods without bone solution in children with OP. We found only one case of lower limb length discrepancy correction with epiphysiodesis in a 14-year-old girl in references [24]. Inan et al. showed that a percutaneous epiphysiodesis can treat limb length discrepancies in children with osteopetrosis.

In our case, genu valgum deformity correction was done by performing medial extraperiosteal hemiepiphysiodesis by tension band technique. Excellent simultaneous limb realignment was achieved in both limbs in 18 months. During preoperative planning, it was impossible to consider the exact rate of angular deformity correction as spontaneous growth of tibia was pathological in OP condition. However, we did not face any difficulties in this regard, probably, because the epiphyseal plates had a good growth rate. The observed speed of correction in the described case was similar to a correction rate in the group of idiopathic knee deformity in the study of Boero et al. [28].

The implant failure was not observed in our patient, but at the moment of material removal one screw was broken because of bone density. It did not compromise the result, but orthopedists should be aware of that eventual complication.

## Conclusion

Thus, we consider the guided growth by tension band technique as a very promising solution for treatment of pediatric angular deformity in patients with OP. This method allows to avoid osteotomy and a related high risk of delayed union or nonunion frequently observed in osteopetrosis. Furthermore, limb realignment in these pathological conditions prevents or slows down the development of secondary osteoarthritis.
